# Mechanisms of nordihydroguaiaretic acid-induced growth inhibition and apoptosis in human cancer cells

**DOI:** 10.1038/sj.bjc.6600186

**Published:** 2002-04-08

**Authors:** T Seufferlein, M J Seckl, E Schwarz, M Beil, G v Wichert, H Baust, H Lührs, R M Schmid, G Adler

**Affiliations:** Department of Internal Medicine I, University of Ulm, D-89081 Ulm, Germany; Department of Radiotherapy, University of Ulm, D-89081 Ulm, Germany; German Cancer Research Center, Applied Tumour Virology, D-69120 Heidelberg, Germany; Department of Medicine, University of Wuerzburg, D-97080 Wuerzburg, Germany; Department of Medical Oncology, CRC Laboratories, Hammersmith Hospitals Campus of Imperial College, London W12 ONN, UK

**Keywords:** NDGA, apoptosis, pancreatic cancer, cervical cancer, JNK, actin cytoskeleton

## Abstract

Lipoxygenase metabolites of arachidonic acid can act as growth promoting factors for various cancer cell lines. Here we demonstrate that the 5-lipoxygenase inhibitor nordihydroguaiaretic acid potently inhibits anchorage-independent growth of human pancreatic and cervical cancer cells in soft agar and delays growth of pancreatic and cervical tumours established in athymic mice. Furthermore, nordihydroguaiaretic acid induces apoptosis of these cancer cells *in vitro* and *in vivo*. Potential mechanisms mediating these effects of nordihydroguaiaretic acid were examined. Nordihydroguaiaretic acid had no inhibitory effect on growth and survival signals such as tyrosine phosphorylation of the epidermal growth factor receptor or basal and growth factor-stimulated activities of extracellular signal-regulated kinase 1/2, p70^s6k^ and AKT but selectively inhibited expression of cyclin D1 in the cancer cells. In addition, treatment with nordihydroguaiaretic acid lead to a disruption of the filamentous actin cytoskeleton in human pancreatic and cervical cancer cells which was accompanied by the activation of Jun-NH_2_-terminal kinase and p38^mapk^. Similar effects were obtained by treatment of the cancer cells with cytochalasin D. These results suggest that nordihydroguaiaretic acid induces anoikis-like apoptosis as a result of disruption of the actin cytoskeleton in association with the activation of stress activated protein kinases. In conclusion, nordihydroguaiaretic acid could constitute a lead compound in the development of novel therapeutic agents for various types of cancer.

*British Journal of Cancer* (2002) **86**, 1188–1196. DOI: 10.1038/sj/bjc/6600186 www.bjcancer.com

© 2002 Cancer Research UK

## 

Arachidonic acid and its metabolites are important second messengers in the signal transduction pathways induced by receptor tyrosine kinases and G protein coupled receptors (Di Marzo, 1995; Rozengurt, 1998). 5-hydrocicosatetraenoic acid (5-HETE), the major 5-lipoxygenase metabolite of arachidonic acid, has been implicated as a growth promoting factor for various human cancer cells including prostate, lung and pancreatic cancer cells (Avis *et al*, 1996; Ghosh and Myers, 1998; Ding *et al*, 1999a). The resinous plant exudate nordihydroguaiaretic acid (NDGA) inhibits lipoxygenases including 5-lipoxygenases (Van Wauwe and Goossens, 1983; Chang *et al*, 1984). Although nordihydroguaiaretic acid (NDGA) may target other kinases (Rondeau *et al*, 1990; Domin *et al*, 1993), it has been used extensively to examine the role of the lipoxygenase pathway in the action of growth factors and cytokines (Haliday *et al*, 1991; Peppelenbosch *et al*, 1992). Indeed, NDGA blocks lipoxygenase production, inhibits growth and induces apoptosis of human lung cancer cells (Avis *et al*, 1996; Ghosh and Myers, 1998). In pancreatic cancer cells, NDGA inhibits thymidine incorporation and anchorage-dependent proliferation and induces apoptosis *in vitro* (Ding *et al*, 1999a,b). However, the potential mechanisms mediating these effects have not been examined. In addition, it is still unclear whether NDGA can block growth of various tumours *in vivo*. This is an important question as numerous compounds inhibit cancer cell growth *in vitro* but fail to be effective *in vivo*.

Apoptosis can be induced by many different events such as direct damage to the cell or its DNA or by the removal of survival signals provided by growth factors, cell–cell contacts and the extracellular matrix. The Raf-MEK-ERK cascade is one of the major signalling pathways promoting cell survival (Parrizas *et al*, 1997; Kurada and White, 1998; Anderson and Tolkovsky, 1999; Bonni *et al*, 1999). However, Ras-dependent cell survival is likely to require additional downstream effectors such as the PI3-kinase-AKT signaling pathway (Datta *et al*, 1999). There is a controversy regarding the contribution of other mitogen-activated protein kinases (MAPKs) such as Jun-NH_2_ terminal kinase (JNKs) and p38^mapk^ to cell survival or apoptosis. Under certain circumstances, activation of JNKs contributes to proliferation. However, in other model systems, activation of JNKs and p38^mapk^ (also known as stress activated protein kinases) can mediate apoptosis (Xia *et al*, 1995; Chen *et al*, 1996; Verheij, 1996; Zanke *et al*, 1996; Bossy-Wetzel *et al*, 1997; Behrens *et al*, 1999; Tournier *et al*, 2000). A similar role for stress activated protein kinases has been proposed for anoikis, the induction of apoptosis in epithelial cells by disruption of cell-cell and cell-matrix contacts (Frisch and Francis, 1994; Frisch *et al*, 1996).

Here we demonstrate that NDGA markedly inhibits growth and induces apoptosis of human pancreatic and cervical cancer cells *in vitro* and *in vivo*. NDGA did not prevent constitutive phosphorylation of p70^s6k^ in these cells which regulates autonomous anchorage-dependent and -independent proliferation of tumour cells (Grewe *et al*, 1999). In addition, NDGA did not inhibit major survival pathways such as tyrosine phosphorylation of the EGFR, or TGFα-induced activation of the ERK cascade and AKT. However, treatment of cells with NDGA leads to activation of JNKs and p38^mapk^, disruption of the filamentous actin cytoskeleton and cell detachment in a sequential fashion. Similar results were observed using cytochalasin D. These results provide novel information about the potential mechanisms of action of NDGA in human pancreatic and cervical cancer cells and suggest that this agent may provide a lead compound for new therapies against both cancers.

## MATERIALS AND METHODS

### Cell culture

Human SW 850 pancreatic (Muller *et al*, 1998; Taniguchi *et al*, 1998) and C4-I cervical cancer cells were the kind gift of W Schmiegel, Department of Internal Medicine, Medical University of Bochum, Germany. Stocks were maintained in RPMI medium supplemented with 10% (v/v) FBS in a humidified atmosphere of 5% CO_2_ : 95% air at 37°C. They were passaged every 3 days.

### Clonogenic Assay

SW 850 and C4-I cells were washed, trypsinised and resuspended in RPMI. Cells were then disaggregated by two passes through a 19-gauge needle into an essentially single cell suspension as judged by microscopy. Cell number was determined using a cell counting chamber and 2×10^4^ cells were mixed with RPMI/0.5% or 10% FBS containing 0.3% agarose in the presence or absence of NDGA at the concentrations indicated, and layered over a solid base of 0.5% agarose in RPMI/0.5% or 10% FBS in the presence or absence of NDGA at the same concentrations in 33 mm dishes. The cultures were incubated in humidified 5% CO_2_ : 95% air at 37°C for 14 days and then stained with the vital stain nitro-blue tetrazolium. Colonies of >120 μm in diameter (25 cells) were counted using a microscope.

### Immunoprecipitations and Western blotting

SW 850 and C4-I cells were washed twice in serum-free RPMI and incubated in fresh RPMI for further 24 h. Cells were then treated with NDGA as indicated in the figure legends and lysed in 50 mM Tris-HCl, 5 mM EDTA, 100 mM NaCl, 40 mM β-glycerophosphate, 50 mM NaF, 1 mM Na_3_VO_4_, 1% Triton X-100, 1 mM phenylmethylsulphonyl fluoride, 10 μg ml^−1^ aprotinin, 10 μg ml^−1^ leupeptin (pH 7.6, lysis buffer). For immunoprecipitations, lysates were incubated with a polyclonal anti-EGF-receptor antibody for 2 h at 4°C on a rotating wheel with protein A sepharose beads added for the second hour. Beads were washed twice in lysis buffer and resuspended in 2× SDS–PAGE sample buffer. Proteins were further analysed by SDS–PAGE followed by Western blotting using a monoclonal anti-Tyr(P) antibody with immunoreactive bands being visualised by enhanced chemoluminescence detection. For detection of cyclin D1, cyclin E, p27^kip1^ and p38 phosphorylated at Thr^180^ and Tyr^182^ and AKT phosphorylated at Thr^308^ cells were treated as indicated in the figure legends, lysed in SDS–PAGE sample buffer and samples were further analysed by SDS–PAGE and Western blotting with specific antisera to these proteins essentially as described above.

### Kinase assays

For ERK assays serum-starved SW 850 and C4-I cells were incubated with TGFα or NDGA in the presence or absence of 15 μM PD 098059 as indicated in the figure legends. Controls received an equivalent amount of solvent. Cells were then lysed at 4°C in 1 ml RIPA buffer (150 mM NaCl, 1% NP-40, 1% DOC, 0.1% SDS, 50 mM Tris, pH 7.4, 2 μg ml^−1^ aprotinin, 40 μg ml^−1^ bestatin, 0.5 μg ml^−1^ leupeptin, 0.5 mM AEBSF, 0.7 μg ml^−1^ pepstatin). Immunoprecipitations were performed at 4°C using an anti-ERK1/2 antibody for 2 h with protein A agarose added for the second hour. Immune complexes were washed three times in lysis buffer and once with ERK kinase buffer (15 mM MgCl_2_, 15 mM Tris-HCl, pH 7.4). Kinase reactions were performed by resuspending the protein A sepharose pellets in 25 μl of kinase assay mixture containing the appropriate kinase buffer with 0.2 μM MBP, 20 μM ATP, 5 μCi ml^−1^ [γ-^32^P]ATP, 2 μM cAMP-dependent protein kinase inhibitor peptide and 100 nM microcystin LR. Incubations were performed under linear assay conditions at 30°C for 20 min and terminated by adding 20 μl of 5×SDS-sample buffer. Samples were boiled for 10 min at 95°C and proteins were separated by SDS–PAGE followed by autoradiography. For JNK and p38^mapk^ cells were washed with PBS and lysed in 20 mM HEPES pH 7.4, 2 mM EGTA, 50 mM β-glycerophosphate, 1 mM DTT, 1 mM Na_3_VO_4_, 10% glycerol, 1% Triton X-100, 2 μM leupeptin, 0.5 mM AEBSF, 5 μg ml^−1^ aprotinin, 0.1 μg ml^−1^ okadaic acid. After 5 min on ice, the lysate was clarified and immunoprecipitated with the respective antibodies. The immunoprecipitates were washed thrice each in lysis buffer and finally in assay buffer (JNK: 20 mM MOPS pH 7.2, 2 mM EGTA, 10 mM MgCl_2_, 1 mM DTT, 0.1% Triton X-100; p38: 20 mM MOPS, pH 7, 1 mM EDTA, 5% glycerol, 0.1% β-mercaptoethanol, 0.01% Brij 35, 0.1 μg ml^−1^ okadaic acid). Kinase reactions contained 20 μl kinase buffer with 1 μg of either ATF2- or cJun-GST fusion protein and Mg-ATP mixes as follows: JNK, 7.5 μl 50 mM MgCl_2_, 100 μM ATP, 2 μCi [γ-^32^P]ATP; p38^mapk^, 6 μl 60 mM Mg acetate, 300 μM ATP and 2 μCi [γ-^32^P]ATP. After 20 min at 30°C, the reactions were stopped by addition of 5×SDS–PAGE sample buffer and further analysed as described above. GST-cJun and GST-ATF2 fusion proteins were prepared essentially as described (Seufferlein *et al*, 1999).

### p70^s6k^ mobility shift assays

Activation of p70^s6k^ by mitogens can be determined by the appearance of slower migrating forms in SDS–PAGE due to phosphorylation of p70^s6k^ on Thr^229^ and Thr^389^ and Ser^404^ which are not phosphorylated in quiescent cells (Ferrari and Thomas, 1994). For p70^s6k^ mobility shift assays cells were treated as indicated in the figure legends and lysed in SDS–PAGE sample buffer. Samples were further analysed by SDS–PAGE and Western blotting with a specific anti-p70^s6k^ antibody.

### **In situ** detection of apoptotic cells

DNA fragmentation was measured by catalytically incorporating fluorescein-12-dUTP at the 3′-OH DNA ends using the enzyme TdT according to the principle of the TUNEL assay. To detect apoptosis in tumour xenografts paraffin-embedded tissue sections of the tumours were deparaffinised and rehydrated through graded ethanol washes, fixed in 4% methanol-free formaldehyde, treated with 20 μg ml^−1^ proteinase K solution for 8–10 min, fixed again and further processed according to the manufacturer's protocol.

### Tumour growth in athymic mice

1.5×10^6^ SW 850 or 2×10^6^ C4-I cells were inoculated subcutaneously into both flanks of 4–6 week old female athymic NMRI/nu-nu mice and the mice were maintained in a pathogen-free environment. The animals were observed daily for tumour development. When measurable tumours were established (>15 mm^3^), the animals were randomised into two groups of six animals. One group received 750 μg NDGA (corresponding to about 90 μM NDGA (w/v)) in 0.1 ml of a solution containing 90% sterile H_2_O and 10% ethanol i.p. 5× per week. The control animals received an equivalent amount of solvent i.p. Tumour size was measured twice weekly and mice were killed after 3 weeks of treatment. Growth curves for xenografts were determined by externally measuring the length, height and width of the tumours, and the volume was calculated according to the following equation: volume=(length×height×width)×0.5. To examine statistical significance an univariate Students *t*-test was performed.

### Materials

NDGA was obtained from FLUKA/Sigma-Aldrich (Deisenhofen, Germany). Antibodies against p70^s6k^, p27^kip1^, cyclin D1, cyclin E, JNK1/2, p38^mapk^, and the EGFR were obtained from Santa Cruz Biotechnology (Santa Cruz, USA). The phospho-specific anti-p38^mapk^ and anti-AKT antibodies were from New England Biolabs (Schwalbach/Taunus, Germany). Protein A sepharose was obtained from Boehringer Mannheim (Mannheim, Germany). ECL-reagent and [γ-^32^P]-ATP were obtained from Amersham/Pharmacia (Freiburg, Germany). The apoptosis detection system fluorescein was from Promega (Mannheim, Germany). Oregon-green labelled phalloidin was obtained from Molecular Probes (Leiden, Netherlands). MBP was from Sigma (Deisenhofen, Germany). All other reagents were of the purest grade available.

## RESULTS

### NDGA delays growth of tumours established in athymic mice

**Figure 1:**
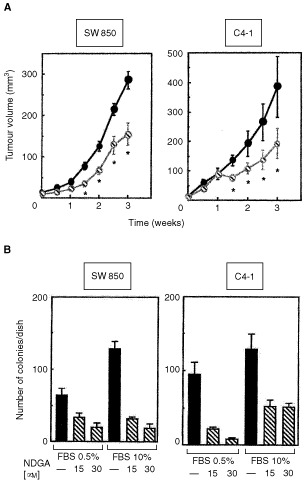
(**A**) NDGA delays growth of xenograft tumours established in athymic mice A: 1.5×10^6^ SW 850 or 2×10^6^ C4-I cells were inoculated subcutaneously into both flanks of 4–6 week old female athymic NMRI/nu-nu mice and the mice were maintained in a pathogen-free environment. When measurable tumours were established (>15 mm^3^), the animals were randomised into two groups of six animals and treated as described in Materials and Methods. Growth curves for xenografts were determined by externally measuring the length, height and width of the tumours at the days indicated. The tumour volume was calculated according to the following equation: volume=(length×height×width)×0.5. Values are the means ±SE (*n*=6). Mice were sacrificed after 3 weeks of treatment with 750 μg NDGA 5× per week i.p. * Indicates a statistically significant difference in tumour size between the NDGA group and the control group (*P*<0.05). (**B**) NDGA inhibits colony growth in SW 850 and C4-I cells. (**A**) Single cell suspensions of SW 850 (left panel), or C4-I cells (right panel) were plated at a density of 3×10^4^ cells/dish in agarose medium containing RPMI and 0.5% (left) or 10% FBS (right) and various concentrations of NDGA as indicated. Colonies were counted after 2 weeks of incubation. In all cases, a representative of two independent experiments each performed in triplicates is shown.

To establish whether NDGA could indeed inhibit tumour growth *in vivo* we first examined a panel of various epithelial cancer cell lines for their ability to consistently induce xenograft tumours in nude mice. Among these tumour cell lines, SW 850 human pancreatic cancer cells and C4-I human cervical cancer cells induced tumour xenografts in athymic mice most consistently and were therefore used for all subsequent experiments (data not shown). Tumours were established by subcutaneous injection of SW 850 and C4-I cells to both flanks of athymic mice. When the tumour volume was 15 mm^3^, mice were treated with five times per week i.p. injections of NDGA or diluent for 3 weeks. As shown in Figure 1A

, treatment with NDGA delayed the growth of both SW 850 and C4-I tumours by about 50%. The compound was very well tolerated by the animals. No side effects or behavioural abnormalities were observed during the course of treatment. There were no marked differences in body weight of the animals treated with NDGA or with solvent during the course of treatment despite the fact that the animals treated with solvent had bigger tumours. In animals injected with SW 850 cells, body weight in the control group was 21.5±1.8 g at the beginning and 24±1.3 g at the end of the treatment. In the NDGA group body weight of the animals was 23.3±1.2 g at the beginning and 24.3±1.4 g at the end of the treatment. The corresponding figures in the animals injected with C4-I cells were 27.5±2.1 g and 29.8±1.7 g in the control group and 27.5±2 g and 29.2±2.1 g in the NDGA group, respectively.

### NDGA inhibits anchorage-independent growth of SW 850 and C4-I cancer cells

In addition to its effect on tumour growth *in vivo* we examined the effect of NDGA on colony formation of SW 850 and C4-I cells in soft agar, a useful *in vitro*-test to judge the efficacy of a compound as a potential anticancer agent (Carney *et al*, 1980). NDGA potently inhibited colony formation in response to both 0.5% FBS and 10% FBS, which induced maximum clonogenic growth in SW 850 and C4-I cells (Figure 1B). Thus, NDGA is a potent inhibitor of anchorage-independent growth in SW 850 and C4-I cells even in the presence of maximum stimulatory concentrations of FBS.

NDGA has previously been reported to inhibit thymidine incorporation and anchorage-dependent proliferation in human pancreatic cancer cells (Ding *et al*, 1999a). We confirmed these observations in SW 850 and also in C4-I cervical cancer cells (data not shown). Interestingly, we observed that as early as 8 h after treatment with NDGA, cells started to detach from the tissue culture dishes making it difficult to determine whether the reduction in thymidine incorporation and cell numbers observed in response to NDGA were due to inhibition of DNA synthesis rather than cell detachment.

### NDGA induces apotosis of SW 850 and C4-I cancer cells *in vitro* and *in vivo*

**Figure 2:**
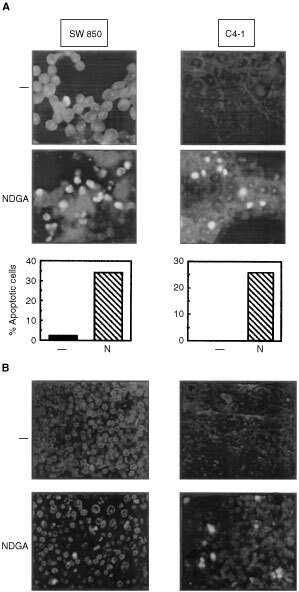
NDGA induces apoptosis in SW 850 and C4-I cells *in vitro* and *in vivo*. (**A**) Upper panels: SW 850 (left) and C4-I cells were incubated with 25 μM NDGA for 18 h and DNA fragmentation was determined by *in situ*-fluorescence TUNEL assays as described in Materials and Methods. Lower panels: The proportion of TUNEL positive cells was counted in four independent microscopic fields (N= NDGA). (**B**) Paraffin sections of tumour xenografts (left SW 850, right C4-I) obtained from mice treated with NDGA (NDGA) or solvent (−) were analysed by an *in situ*-fluorescence TUNEL assay to detect DNA fragmentation as described in Materials and Methods. Apoptotic cells are characterised by a strong fluorescence within the nucleus corresponding to labelling of fragmented DNA.

NDGA has been reported to induce apoptosis in certain cancer cells *in vitro*. Indeed, NDGA induced apoptosis in SW 850 pancreatic cancer cells and also in C4-I human cervical cancer cells as judged by TUNEL assays (Figure 2A

). Upon treatment of cells with 25 μM NDGA for 12 h, the proportion of cells exhibiting fragmented DNA markedly increased from 2 to 34% in SW 850 and from 0 to 26% in C4-I cells. The apoptosis-inducing effect of NDGA was first detectable after 3 h of incubation and reached a maximum after 12–16 h of incubation (data not shown). To examine whether NDGA induced similar molecular mechanisms in the pancreatic and cervical xenograft tumours *in vivo*, paraffin-embedded tissue sections of tumours obtained from animals treated with NDGA or with solvent were examined by *in situ*-fluorescence TUNEL assays. Treatment of mice with NDGA led to a marked increase in the number of apoptotic cells in tumours established from SW 850 and C4-I cells (Figure 2B). Thus, NDGA induces apoptosis in pancreatic and cervical tumours *in vitro* and *in vivo*.

### NDGA does not inhibit growth and survival signals in SW 850 and C4-I cells

**Figure 3:**
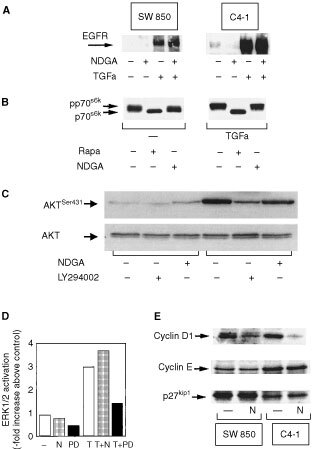
(**A**) TGFα-induced tyrosine phosphorylation of the EGFR in SW 850 and C4-I cells is not affected by NDGA treatment of cells. Serum starved SW 850 (left) or C4-I cells (right) were treated for 60 min with 25 μM NDGA (+). Control cells received an equivalent amount of solvent (−). Cells were subsequently treated with 50 ng ml^−1^ TGFα (TGFα, +) or an equivalent amount of solvent (−) for 10 min. Cells were then lysed and tyrosine phosphorylation of the EGFR was further analysed as described in Materials and Methods. (**B**) NDGA does not inhibit constitutive and TGFα-stimulated phosphorylation of p70^s6k^ in SW 850 cells. Serum-starved cultures of SW 850 cells were treated for 15 min with 50 ng ml^−1^ TGFα in the absence (−) or presence of 30 μM NDGA (NDGA, +) or 20 ng ml^−1^ rapamycin (Rapa, +) or received an equivalent amount of solvent (−). p70^s6k^ mobility shift assays were performed as described in Materials and Methods. The results shown in each case are representative of three independent experiments. The positions of hypophosphorylated p70^s6k^ (p70^s6k^) and the slower migrating phosphorylated p70^s6k^ (pp70^s6k^) are indicated by arrows. (**C**) NDGA does not inhibit basal and TGFα-stimulated activation of AKT. Serum-starved cultures of C4-I cells were treated for 15 min with 50 ng ml^−1^ TGFa in the absence (−) or presence of 30 μM NDGA (+) or 20 μM LY294002 (+) or received an equivalent amount of solvent (−). Phosphorylation of AKT at Ser^431^ was determined using an activation-specific antibody as described in Materials and Methods and is indicated by an arrow (AKT^Ser431^). The same samples were also analysed using a pan-AKT antibody. The position of AKT is indicated by an arrow. (**D**) NDGA does not inhibit basal and TGFα-stimulated activation of ERK1/2: Serum-starved cultures of C4-I cells were treated for 15 min with 50 ng ml^−1^ TGFα (T) in the absence (−) or presence of 25 μM NDGA (N) or 15 μM PD 098059 (PD) or received an equivalent amount of solvent (−). ERK1/2 immune complex kinase assays were performed as described in Materials and Methods. (**E**) NDGA inhibits constitutive cyclin D1 expression in SW 850 and C4-I cancer cells: Subconfluent cultures of SW 850 and C4-I cells were treated with 25 μM NDGA (N) for 18 h. Control cells received an equivalent amount of solvent (−). Cells were lysed and further analysed by Western blotting with either anti-cyclin D1, anti-cyclin E or anti-p27^kip1^ antibodies as indicated by an arrow. The results shown in each case are representative of at least three independent experiments.

Next, we were interested in potential mechanisms mediating the growth inhibitory effect of NDGA in human pancreatic and cervical cancer cells. NDGA has been shown to inhibit tyrosine phosphorylation of certain receptor tyrosine kinases (Domin *et al*, 1993). The EGFR and its respective ligands such as TGFα trigger growth and prevent apoptosis by autocrine and/or paracrine mechanisms in many cancer cell lines (Korc *et al*, 1992; Bonni *et al*, 1999; Seufferlein *et al*, 1999). To examine whether NDGA could inhibit tyrosine phosphorylation of the EGFR, serum starved SW 850 and C4-I cells were treated with TGFα in the absence or presence of NDGA and autophosphorylation of the EGFR was determined by anti-Tyr(P) Western blotting. NDGA had no effect on TGFα-induced tyrosine phosphorylation of the EGFR in SW 850 and C4-I cells, respectively (Figure 3A

). Similar data were obtained for insulin-like growth factor I-induced phosphorylation of the IGF-I receptor (data not shown).

Activity of the serine-threonine kinase p70^s6k^ is important for progression from the G1 to the S phase of the cell cycle (Chou and Blenis, 1995). We have recently demonstrated that the FRAP-p70^s6K^ pathway is constitutively active in human pancreatic cancer cells and regulates autonomous growth of these cells (Grewe *et al*, 1999). As shown in Figure 3B, NDGA did not substantially inhibit basal phosphorylation of p70^s6k^. NDGA treatment of SW 850 cells also did not markedly interfere with further phosphorylation of p70^s6k^ in response to TGFα. In contrast, treatment of cells with the selective inhibitor of the FRAP-p70^s6k^ pathway, rapamycin (Brown *et al*, 1995), prevented both, basal and TGFα-stimulated phosphorylation of p70^s6k^ (Figure 3B). Similar results were obtained in the C4-I cell line (data not shown).

Ras activates two major survival pathways, the ERK cascade and AKT (Anderson and Tolkovsky, 1999; Bonni *et al*, 1999; Datta *et al*, 1999). NDGA did not inhibit phosphorylation of the antiapoptotic protein kinase AKT in TGFα-stimulated C4-I cells. In contrast, inhibition of the major upstream regulator of AKT, PI3-kinase, by the selective PI3-kinase inhibitor LY294002 markedly prevented TGFα-stimulated AKT phosphorylation in C4-I cells (Figure 3C). Similar data were obtained in SW 850 cells (data not shown). NDGA had also no effect on basal and TGFα-stimulated activation of ERK1/2. In contrast, the selective MEK-1 inhibitor PD 098059 inhibited both basal and TGFα-stimulated activation of ERK1/2 (Figure 3D). These data demonstrate that NDGA does not act as an inhibitor of the EGFR kinase or the activation of the Ras/Raf/ERK- or the Ras/PI3-kinase/AKT/p70^s6k^ signalling pathways in pancreatic and cervical cancer cells.

### NDGA inhibits expression of cyclin D1 in SW 850 and C4-I human cancer cells

Thus, inhibition of tumour cell growth by NDGA could not be explained by inhibition of the major growth promoting intracellular signalling pathways in response to NDGA. Therefore, we reasoned that this compound could directly affect the regulation of the cell cycle machinery. In particular, cyclin D1 is a major regulator of proliferation at the level of the cell cycle (Sherr, 1993) and is overexpressed in many cancers (Weinstein, 1996). In pancreatic cancer, overexpression of cyclin D1 is associated with increased aggressiveness of these tumours (Kornmann *et al*, 1998). We have previously shown that cyclin D1 and E but also cyclin-dependent kinase inhibitors such as p27^kip1^ are constitutively expressed in human pancreatic cancer cells (Grewe *et al*, 1999). As shown in Figure 3E, cyclin D1, cyclin E and p27^kip1^ were also constitutively expressed in C4-I human cervical cancer cells. Incubation of cells with NDGA resulted in a marked reduction in the expression of cyclin D1 in both cell lines whereas the levels of expression of cyclin E and p27^kip1^ remained unchanged. These results suggest that the inhibition of proliferation in response to NDGA in SW 850 and C4-I cells could, at least in part, be mediated by inhibition of cyclin D1 expression.

### NDGA and cytochalasin D induce depolymerisation of the actin cytoskeleton in SW 850 and C4-I cells

**Figure 4:**
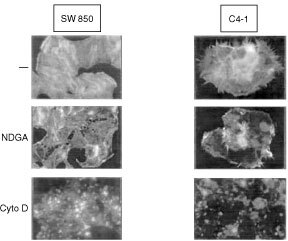
NDGA and cytochalasin D induce breakdown of the actin cytoskeleton. Serum starved SW 850 (left) and C4-I cells (right) were treated with 25 μM NDGA (NDGA) or 2 μM cytochalasin D (Cyto D) for 60 min or received an equivalent amount of solvent (−). Cells were subsequently fixed and actin was further analysed by immunocytochemistry as described in Materials and Methods.

The actin cytoskeleton in concert with adhesion molecules controls cell-cell and cell-substrate interactions and participates in transmembrane signalling. Upon treatment of cells with NDGA we observed substantial cell detachment. This could be a consequence of the induction of apoptosis, but also due to a more direct effect of NDGA on the cytoskeleton in pancreatic and cervical cancer cells. To examine the effect of NDGA on the actin cytoskeleton in SW 850 and C4-I cells, actin was analysed by immunofluorescence using Oregon-green-labelled phalloidin after treatment of cells with NDGA or solvent. As shown in Figure 4

(top panels), control cells exhibited a well developed actin cytoskeleton with a circumferential actin filament network, actin microspikes at the plasma membrane and actin stress fibres crossing the cells. Strikingly, within 30 min of exposure to 25 μM NDGA, actin stress fibers disappeared and the number of mikrospikes was markedly reduced. A maximum effect of NDGA on the actin cytoskeleton was observed after 60 min of incubation (Figure 4, middle panels) and clearly preceded cell detachment and the induction of apoptosis in response to NDGA (data not shown). Interestingly, cortical actin filaments were less affected by the NDGA treatment (Figure 4, middle panels). The effect of NDGA on the filamentous actin cytoskeleton was comparable to that of cytochalasin D. However, in addition to the disruption of actin stress fibres, cytochalasin D also disrupted the circumferential actin filament network (Figure 4, bottom panels). The effect of cytochalasin D on the actin cytoskeleton in SW 850 and C4-I cells was first visible at 0.3 μM and reached a maximum at 2 μM cytochalasin D. At these concentrations, cytochalasin D also induced apoptosis in both cell lines (data not shown). These data suggest that NDGA by inhibiting the organisation of actin stress fibers in SW 850 and C4-I cells interferes with cell-matrix interaction and induces anoikis.

### NDGA and cytochalasin D activate stress-activated protein kinases in SW 850 and C4-I cells

**Figure 5:**
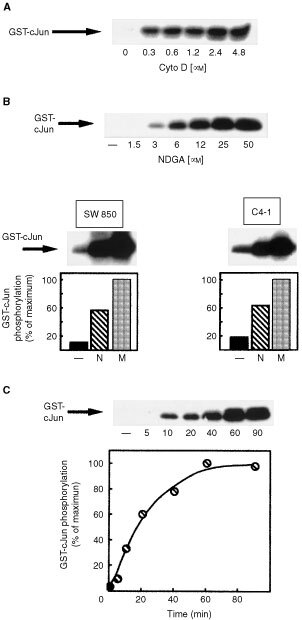
(**A**) Cytochalasin D activates of JNK1/2 in SW 850 and C4-I cells: Top panel: SW 850 cells were incubated with various concentrations of cytochalasin D (Cyto D) for 60 min as indicated. Control cells received an equivalent amount of solvent. JNK activity was determined in JNK-immune complex kinase assays using a GST-cJun fusion protein as substrate as described in Materials and Methods. The position of the GST-cJun fusion protein is indicated by an arrow. (**B**) NDGA induces activation of JNK1/2 in SW 850 and C4-I cells. Top panel: Concentration dependence of NDGA-induced JNK activation. C4-I cells were incubated with various concentrations of NDGA for 60 min as indicated. Control cells received an equivalent amount of solvent. Cells were further analysed by JNK1/2-immune complex kinase assays using GST-cJun as substrate. Middle and bottom panels: SW 850 (left) and C4-I cells (right) were incubated with 25 μM NDGA (N) or 1 mM mannitol (M) for 60 min or received an equivalent amount of solvent (−). Cells were subsequently analysed by JNK-immune complex kinase assays as described in Materials and Methods. The experiments shown are representative of at least three independent experiments. Results from a representative experiment were subjected to scanning densitometry. The data are expressed as per cent of maximal GST-cJun phosphorylation obtained in response to 1 mM mannitol in both cell lines. (**C**) Kinetics of NDGA-induced activation of JNK1/2. Top: Serum starved SW 850 cells were treated with 25 μM NDGA for various times as indicated and JNK1/2-immune complex kinase assays were performed as described in Materials and Methods. A typical result in SW 850 cells was subjected to scanning densitometry. Data are expressed as per cent of maximal JNK 1/2 activation obtained after 60 min of incubation.

Anoikis by detachment of epithelial cells from their matrix is associated with the activation of Jun-NH_2_-terminal kinases (Frisch *et al*, 1996). Treatment of SW 850 and C4-I cells with cytochalasin D also induced JNK1/2 activation in a concentration dependent manner reaching a maximum at 2.4 μM cytochalasin D in both cell lines (Figure 5A

and data not shown). Interestingly, NDGA also induced a dose dependent activation of JNK1/2 in both cell lines with a maximum effect at 25 μM (Figure 5B, top panel and data not shown). In both cell lines, GST-cJun phosphorylation in response to NDGA was about 60% of that in response to 1 mM mannitol, a potent osmotic stressor (Kyriakis and Avruch, 1996) (Figure 5B, bottom panels). Furthermore, NDGA induced phospho-JNK immunoreactivity exclusively in the nucleus of SW 850 and C4-I cells treated with NDGA providing additional evidence that NDGA is a strong activator of JNKs (Cavigelli *et al*, 1995; Sanchez-Perez *et al*, 1998; data not shown).

Figure 5C shows the kinetics of NDGA-induced JNK1/2 activation. Activation of JNK1/2 in response to NDGA was detectable as early as 10 min following treatment of SW 850 and C4-I cells with 25 μM NDGA; a maximum effect was obtained after 60 min of incubation.

**Figure 6:**
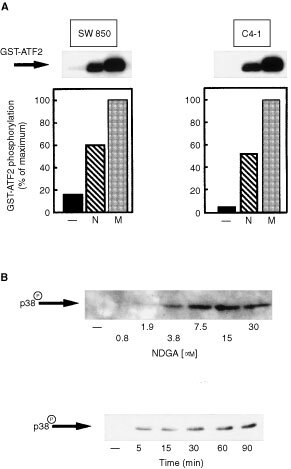
NDGA selectively stimulates p38^mapk^ activation in SW 850 and C4-I cells. (**A**) Top: SW 850 and C4-I cells were incubated with 25 μM NDGA (N) or 1 mM mannitol (M) for 60 min. Control cells received an equivalent amount of solvent (−). Cells were further analysed by p38^mapk^ immune complex kinase assays using a GST-ATF2 fusion protein as substrate. (**A**) Bottom panels: Typical results of p38^mapk^ immune complex kinase assays in SW 850 (left) and C4-I cells (right) were subjected to scanning densitometry. Data are expressed as per cent of maximal GST-ATF2 phosphorylation obtained in response to 1 mM mannitol. (**B**) Top panel: Serum starved SW 850 cells were incubated with various concentrations of NDGA for 60 min and further analysed by Western blotting using a phosphospecific anti-p38^mapk^ antibody as described in Materials and Methods. (**B**) Bottom panel: Serum starved C4-I cells were incubated with 25 μM NDGA for various times and further analysed by Western blotting using a phosphospecific anti-p38^mapk^ antibody as described in Materials and Methods.

NDGA did not affect the activation of the non-stress related members of the MAPK family ERK1/2 in cancer cell lines examined. In marked contrast, the results in Figure 6A

demonstrate that NDGA activates the stress related p38^mapk^ in immune complex kinase assays reaching 60 and 50% of mannitol-induced GST-ATF2 phosphorylation in SW 850 and C4-I cells, respectively. Activation of p38^mapk^ occurs upon dual phosphorylation at Thr^180^ and Tyr^182^ (Han *et al*, 1994). Using an antibody which specifically detects phosphorylation of the kinase at these two residues, p38^mapk^ phosphorylation was first detectable at about 2 μM NDGA reaching a maximum at 15 μM NDGA in C4-I and SW 850 cells (Figure 6B, top panel and data not shown). p38^mapk^ phosphorylation could be detected as early as 5 min after incubation with NDGA reaching a maximum after 30 min of incubation (Figure 6B, bottom panel and data not shown). Thus, NDGA selectively activates the stress-activated protein kinases of the MAPK family.

## DISCUSSION

A better understanding of the biology of tumours could greatly improve our current concepts of cancer therapy. Arachadonic acid and its metabolites such as 5-HETE have been implicated as growth promoting factors for various human cancers (Avis *et al*, 1996; Ghosh and Myers, 1998; Ding *et al*, 1999a). Here, we demonstrate that the resinous plant exsudate NDGA, a 5-lipoxygenase inhibitor, markedly inhibits anchorage-independent growth of human pancreatic and cervical cancer cells in serum as well as growth of xenograft tumours established from these cells in athymic mice. NDGA did not interfere with major growth promoting signaling pathways such as the activation of receptor tyrosine kinases, PI3-kinase/p70^s6k^ or the ERK cascade demonstrating that NDGA is not acting as a general kinase inhibitor in pancreatic and cervical cancer cell lines. Many cancers have been shown to lose growth control as a result of defects in cell cycle control. Cyclin D1, which is crucial for promoting cell cycle progression, is constitutively expressed in pancreatic (Kornmann *et al*, 1998), but also in cervical cancer cells and could – at least in part – be responsible for the transformed phenotype of these cells. Our data demonstrate that NDGA selectively inhibits constitutive cyclin D1 expression in both cancer cell lines providing a molecular mechanism of its growth inhibitory properties.

Inhibition of cyclin D1 can lead to cell cycle arrest and under certain circumstances to the induction of apoptosis (Tan *et al*, 2000). NDGA markedly induced apoptosis in SW 850 and C4-I cells *in vitro* and also in xenograft tumours established in athymic mice. This suggests that similar mechanisms mediate the effects of NDGA *in vitro* and *in vivo*. Again, the induction of apoptosis by NDGA was not due to inhibition of Ras-dependent survival pathways including the antiapoptotic kinase AKT. Instead, our data suggest that NDGA induces anoikis by disrupting the filamentous actin cytoskeleton in human pancreatic and cervical cancer cells. The effect of NDGA on actin stress fibers was comparable to that of cytochalasin D. In contrast to cytochalasin D, the effect of NDGA on the actin cytoskeleton appears to be cell type specific: NDGA could affect actin stress fibres in fibroblasts (Hirata *et al*, 1984; Chong *et al*, 1987), but failed to do so in leukocytes (Shalit *et al*, 1987) or keratinocytes (Coutant *et al*, 1997).

Anoikis is accompanied by induction of stress activated protein kinases (Frisch *et al*, 1996; Khwaja and Downward, 1997). Indeed, cytochalasin D potently induced activation of JNK1/2 in both cell lines. NDGA similarly activated JNK1/2 and p38^mapk^ but had no effect on ERK1/2 activation in SW 850 and C4-I cells. Again, this effect of NDGA is likely to be cell type specific: No effect of NDGA on JNK activation could be demonstrated in vascular smooth muscle cells (Madamanchi *et al*, 1998), HeLa cells and HL 60 cells (Hii *et al*, 1998). There is some controversy regarding the possible role of stress activated protein kinases as mediators of apoptosis. For example, there is evidence that these kinases can mediate apoptosis under certain circumstances (Behrens *et al*, 1999; Tournier *et al*, 2000) including anoikis (Frisch *et al*, 1996). However, it has been questioned whether JNK activation occurring 30 min after suspending the cells by trypsinisation can contribute to anoikis (Khwaja and Downward, 1997). The sequence of events presented in this manuscript is different: We show that disruption of the actin cytoskeleton by NDGA is associated with activation of JNK and p38^mapk^ and that both events precede cell detachment. The early activation of JNKs and p38^mapk^ prior to cell detachment could contribute to anoikis. Our finding that similar concentrations of NDGA are required for disruption of actin stress fibres, JNK activation and the induction of apoptosis further supports our conclusion that these events could be related. However, the precise contribution of JNK1/2 and p38^mapk^ activation to NDGA-induced cytoskeletal disruption and apoptosis requires further examination.

NDGA has been widely used as a specific lipoxygenase inhibitor. The precise relationship between the inhibitory effect of NDGA on lipoxygenases and the signalling events induced by NDGA described in this paper is not clear. Ding *et al* (1999a) described an inhibitory effect of NDGA on basal and 5-HETE-induced DNA synthesis in pancreatic cancer cells at concentrations comparable to those used in our experiments. This could suggest that some of the signalling events are related to the lipoxygenase inhibitory action of NDGA. However, the activation of JNKs and p38^mapk^ in response to NDGA seems to be cell type specific, whereas NDGA will inhibit lipoxygenases in virtually all cells. Thus, either the consequences of lipoxygenase inhibition are cell type specific or NDGA induces additional effects in a cell-type specific manner which are independent from its lipoxygenase inhibitory actions. Therefore, given the activation of multiple signalling events by NDGA, this compound should not be used anymore as a ‘specific’ lipoxygenase inhibitor without the examination of additional pathways.

The effect of NDGA on tumour growth observed *in vivo* was moderate but there was no toxicity detectable in the animals at the concentration used. The fact that NDGA seems to be effective against diverse and often incurable tumour types suggests that a more detailed analysis of the effects of NDGA on tumour growth *in vivo* is urgently required. Moreover, NDGA could provide a lead compound for the development of novel therapeutics in pancreatic and cervical cancer.
